# Factors Influencing Immune Restoration in People Living with HIV/AIDS

**DOI:** 10.3390/jcm11071887

**Published:** 2022-03-28

**Authors:** Bogusz Jan Aksak-Wąs, Anna Urbańska, Kaja Scheibe, Karol Serwin, Magdalena Leszczyszyn-Pynka, Milena Rafalska-Kosior, Joanna Gołąb, Daniel Chober, Miłosz Parczewski

**Affiliations:** Department of Infectious, Tropical Diseases and Immune Deficiency, Pomeranian Medical University in Szczecin, 71-455 Szczecin, Poland; urbanska@pum.edu.pl (A.U.); kaja.scheibe@pum.edu.pl (K.S.); karolserwin@gmail.com (K.S.); mlpynka@interia.pl (M.L.-P.); mil.rafalska@gmail.com (M.R.-K.); asiak119@interia.pl (J.G.); kalendrial@gmail.com (D.C.); mparczewski@yahoo.co.uk (M.P.)

**Keywords:** immune restoration, PLWHA, CCR2 (rs1799864), CCR5-Δ32 (rs333), HLA-B*5701

## Abstract

Introduction: Immune restoration is a key clinical aspect that is pursued in the care of human immunodeficiency virus (HIV)-infected patients. Despite effective antiretroviral treatment and undetectable viremia, immune recovery is often incomplete. Materials and methods: Data from 311 Caucasian patients were collected. SNP in CCR2(rs1799864), CX3CR1(rs3732378), HLAC-35(rs9264942), and CCR5(promoter, rs1799988); a 32bp deletion(Δ32) in CCR5; and HLA-B*5701 genotypes were correlated with clinical data and selected endpoints. Kaplan–Meier and Cox proportional hazards models were used to analyze the effects of genetic factors over time. Results: For HLA-B*5701, the effect on the CD4+/CD8+ >0.8 cell ratio was lost within 48 months (HR = 2.04, 95% CI: 1.04–4.03), and the effect on the CD4+ cell count >500 cells/µL was lost within 12 months (HR = 2.12, CI: 1.11–4.04). The effect of CCR2 GG on the CD4+/CD8+ >0.8 cell ratio was lost within 36 months (HR = 1.7, CI: 1.05–2.75). For CCR5 wt/Δ32, the effect on the CD4+/CD8+ >1.0 cell ratio was lost within 24 months (HR = 2.0, CI: 1.08–3.69), and the effect on the CD4+ >800 cells/µL cell count was lost within 18 months (HR = 1.98, CI: 1.14–4.73). Conclusions: Selected genetic polymorphisms, namely CCR2 GG and CCR5 Δ32, and the presence of the HLA-B*5701 allele positively influenced immune restoration in cART-treated patients with HIV/AIDS.

## 1. Introduction

Studies of the natural history of HIV infection estimated that during the first 8 years from transmission, viremia increases by approximately 0.04 log copies/mL/year [1]. It is estimated that almost all PLWHA will experience the progression of infection if left untreated, with the exception of the so-called “elite controllers” and “viremic controllers”, who constitute special cases. Elite controllers are patients who maintain an undetectable HIV viral load, despite being infected, and viremic controllers are patients with a viral load not exceeding 50–2000 copies/mL, despite the lack of antiretroviral treatment [2]. The typical rate of decrease in the number of CD4+ lymphocytes during HIV infection is 30-40/µL/year. This is true even among elite controllers with undetectable viremia. However, on average, only approximately 50% of elite controllers have a CD4+ lymphocyte count > 500/µL at the time of diagnosis [1].

The reversion of immunodeficiency is achieved with effective antiretroviral therapy. Immune restoration is the key goal that is pursued in the care of HIV-infected patients. Despite proper treatment and undetectable viremia, the patient’s immune recovery may not be complete. There are significant differences in both the recovery of CD4+ lymphocytes and the maintenance of their normal numbers depending on age and comorbidities, and these factors affect mortality risk [3]. Relatively few people achieve immune recovery based on the number of CD4+ lymphocytes in their peripheral blood or lymph nodes. Up to 20% of patients may experience immune failure, despite the suppression of HIV infection, with or without limited increases in the number of CD4+ T cells [4,5,6].

Host genetic variants (specifically in the human leukocyte antigen (HLA) and chemokine genes) strongly influence the outcome of HIV-1 infection, the degree of immune activation, the extent of acute viremia, and the magnitude of the latent viral reservoir. An array of variants is previously shown to be associated with lower baseline HIV viral load and a slower decline in the number of CD4+ T-cells [7]. In this study, variants in the CCR2 (rs1799864), CCR5 (promoter, rs1799988), CX3CR1 (rs3732378), HLAC-35 (rs9264942), CCR5 (Δ32, rs333), and HLA-B (*5701) genes were selected for analysis. These genetic variants were selected due to the previously demonstrated significance of these factors in HIV infection, both in viremic and immune contexts. CCR2 variants are associated with a protective effect against the progression of HIV infection, with a 58% lower risk of developing AIDS in the first 4 years after seroconversion and a 19% lower risk during the next 4 years of observation after seroconversion [8]. These polymorphisms are also associated with a reduction in mortality in vertically infected children during the first years of their lives [9].

The rs1799988 variant in the CCR5 promoter influences the progression of HIV. Its effect consists of modifying the expression levels of CCR5 receptors on the surface of CD4+ cells, which affects the ability of HIV [10] to infect other cells. The A allele of the CX3CR1 variant (rs3732378) may limit the shift in HIV-1 tropism from CCR5 to CXCR4. This effect may be related to the amount of co-receptor on the cell surface. Polymorphisms of this chemokine receptor gene may influence disease progression and HIV tropism [11]. In addition, a 32 base pair deletion in the CCR5 gene is associated with a slower progression of HIV infection and a positive effect on survival among patients not treated with combination antiretroviral therapy (cART). Some studies report that CCR5-Δ32 is the most potent protective variant, in both immunological and viremic contexts, unrelated to HLA [9,12]. The HLA-C promoter variant (rs9264942) is associated with a significant reduction in HIV viral load in the untreated population, especially in the context of CC homozygosity [13]. The reduction in baseline HIV viral load may be related to the expression levels of HLA-C mRNA and membrane receptors, which depend on the presence of the T allele [12,13,14,15,16,17,18,19]. Finally, the HLA-B*5701 variant, which is currently extensively used in clinical practice because of the associated abacavir hypersensitivity [20,21,22], is widely associated with a significant protective effect by inhibiting HIV replication and delaying infection progression [23,24].

The aim of this study was to assess the impact of selected genetic variants on immune recovery in HIV-positive individuals. We attempted to determine the influence of both clinical and genetic variables on immune restoration.

## 2. Materials and Methods

### 2.1. Study Population

Longitudinal data were collected from 834 Caucasian patients who were followed up at the Department of Acquired Immunodeficiency, Pomeranian Medical University, Szczecin, Poland. The study protocol was approved by the Bioethical Committee of Pomeranian Medical University (approval number BN-001/34/04). Entry into care was taken as the date of the first positive HIV test, if confirmed by immunoblotting or serum HIV RNA detection. The following data were collected: age, sex, route of transmission, HIV subtype (known for 207 patients), clinical diagnosis category according to the Centers for Disease Control and Prevention (CDC 1993 [25]), case definition, baseline HIV viral load, and baseline CD4+ lymphocyte count. The baseline CD4+ lymphocyte count was defined as the first documented result after the diagnosis of HIV infection, regardless of the number of lymphocytes.

Patients were included in the study if they showed effective antiretroviral treatment, as defined by undetectable viremia (<50 HIV RNA copies/mL) within 6 months from the initiation of antiretroviral treatment. Antiretroviral treatment was initiated in patients in accordance with the guidelines of the European AIDS Clinical Society (EACS) and Polish AIDS Society (PTN AIDS), valid for the year of inclusion, verified depending on the changing guidelines. They were based on a triple-drug therapy (2×NRTI (nucleoside/-tide reverse transcriptase inhibitors) + INSTI (integrase strand transfer inhibitors)/PI+b (Protease inhibitors+ booster)/NNRTI (non-nucleoside reverse-transcriptase inhibitors)). In recent years, when dual-drug therapy was approved, single patients were treated on the basis of dual-drug regimens. Additionally, patients were required to maintain undetectable viremia throughout the observation period, with an allowance for one viral load polymerase chain reaction (PCR) test showing >200 copies/mL during each year of observation. Cases with repeatedly detectable viral load during the observation period or adherence <90%, as assessed by the treating physician, were excluded from the dataset.

Following a review of the dataset, 311 patients met all of the selection criteria and were included in the final analysis. Of these, 225 were men (72.3%) and 86 were women (27.7%). During the entire observation period, 16 patients died, accounting for 5.14% of the study group.

### 2.2. Immune Recovery Analyses

As there is no clear consensus which criterium signifies immune recovery, different criteria were assessed in the current study, i.e., restoration to 500 and 800 CD4+ lymphocytes/µL of blood and the restoration of the CD4+/CD8+ cell ratio (both >0.8 and >1.0 ratios) [26,27,28]. The combination of a CD4 lymphocyte count >500 and CD4/CD8 ratio >1 was considered the primary endpoint of immune restoration.

The maximum observation period was 120 months. 

### 2.3. DNA Extraction and Genotyping

The following single nucleotide polymorphisms (SNPs) and genetic variants were analyzed in this study: rs1799864 in CCR2, rs1799988 in the CCR5 promoter, rs3732378 in CX3CR1, rs9264942 in HLAC-35, a 32 base pair deletion (Δ32) in the coding region of CCR5 (rs333), and HLA-B*5701. Genomic DNA was extracted from whole blood samples using a DNA Blood Mini Kit (Qiagen, Hilden, Germany) and stored at 4 °C for further analysis. If whole blood samples were unavailable, DNA was extracted from frozen serum samples using the Sherlock AX kit (A&A Biotechnology, Gdynia, Poland).

SNPs (rs1799864, rs1799988, rs3732378, and rs9264942) were analyzed using validated SNP Genotyping Assay reagents and the Genotyping Master Mix (Life Technologies, Carlsbad, CA, USA). The sequences of the primers and probes were not disclosed by the manufacturer. Amplification was performed using a StepOne device (Applied Biosystems, Foster City, CA, USA) under standard reaction conditions, as stipulated by the assay manufacturer. 

HLA-B*5701 genotyping was performed using sequence-specific PCR (SS-PCR) with a HLA-Ready Gene B5/57 low-resolution kit (Inno-Train Diagnostik, Kronberg, Germany), according to the manufacturer’s protocol. This assay is validated for in vitro diagnostic use and has Conformite Europeenne (CE) certification. Positive samples were verified using another CE-certified assay, the Olerup SSP HLA-B* 57 high-resolution kit (Olerup SSP AB, Saltsjoebaden, Sweden), with subsequent electrophoresis [20]. The CCR5-Δ32 variant was detected by SS-PCR, as previously described [29]. All SS-PCR reaction products were electrophoresed on 3% agarose gels stained with DNA-star dye (Lonza, Basel, Switzerland) and visualized under ultraviolet light. 

HIV RNA quantification was performed using either the Real-Time HIV-1 test from Abbott Molecular (Des Plaines, IL, USA) or the Cobas AmpliPrep/Cobas TaqMan HIV-1 test manufactured by Roche Molecular Systems, Inc. (Mannheim, Germany), based on availability. Meanwhile, the lymphocyte CD4+ cell count was determined using standard validated assays from Becton–Dickinson (Franklin Lakes, NJ, USA).

### 2.4. Statistical Analysis

Statistical analyses were performed for nominal variables using a chi-square test (sex, HIV infection stage at genotyping, transmission route), while continuous variables (age at HIV diagnosis, CD4+ lymphocyte count, HIV viral load at care entry) were analyzed using a Mann–Whitney U-test (Statistica v12 software; Statsoft, Tulsa, OK, USA). Statistical significance was set at *p* < 0.05.

For individual SNPs, the frequencies of both genotypes and individual alleles were calculated. The Hardy–Weinberg equilibrium was assessed, and the significance of the deviation from the expected frequencies of a given variant was calculated using a chi-square test. Data were collected from patients at the time of diagnosis or when they entered care. Time zero was defined as the time of a positive HIV infection result, as confirmed by a western blotting test, or, if not available, the date of entering care at the outpatient clinic.

For individual parameters, the Cox proportional hazard coefficients were assessed collectively. Clinical statistics were censored at the 120-month endpoint. Genetic data were censored at 60 months (due to a lack of statistical significance after this timepoint), with analysis every 6 months (Table 1). Only data with significant differences are shown.

Every genetic variant was tested at the 12-month timepoint, as this timepoint was shown to have the largest hazard ratio of immune recovery. In addition to the Cox proportional hazard assessment, a log-rank analysis of the Kaplan–Meier estimator for immune restoration was performed at selected timepoints.

## 3. Results

### 3.1. Patient Characteristics

Men comprised the majority of the study group (*n* = 225; 72.3%), which had an average age of 33 years (interquartile range IQR: 27–40). The distribution of the routes of infection was as follows: men having sex with men, 113 (42.3%); heterosexual contact, 98 (36.7%); and intravenous drug use, 56 (21.0%). The median baseline number of CD4+ lymphocytes/µL was 301 (IQR: 107–481). The baseline viral load was 4.96 log (IQR: 4.26–5.53) copies/mL. The total observation time for the analyzed cohort was 2559.4 patient years. 

The number of patients with immune reconstitution to CD4+ lymphocyte counts >500 cells/μL was 262 (84.2%) within a median (IQR) of 9.28 (2.93–24.87) months, while 145 (46.6%) patients recovered to CD4+ lymphocyte counts >800 cells/μL, within a median (IQR) of 23.4 (9.21–52.01) months. Furthermore, 194 (62.4%) patients showed a restored CD4+/CD8+ cell ratio >0.8 within a median (IQR) of 17.53 (4.9–45.07) months, and 142 (45.7%) showed a CD4+/CD8+ cell ratio >1.0, within a median (IQR) of 24.93 (9.44–61.68) months.

### 3.2. Genetic Factors and Immune Restoration 

**Figure 1:**
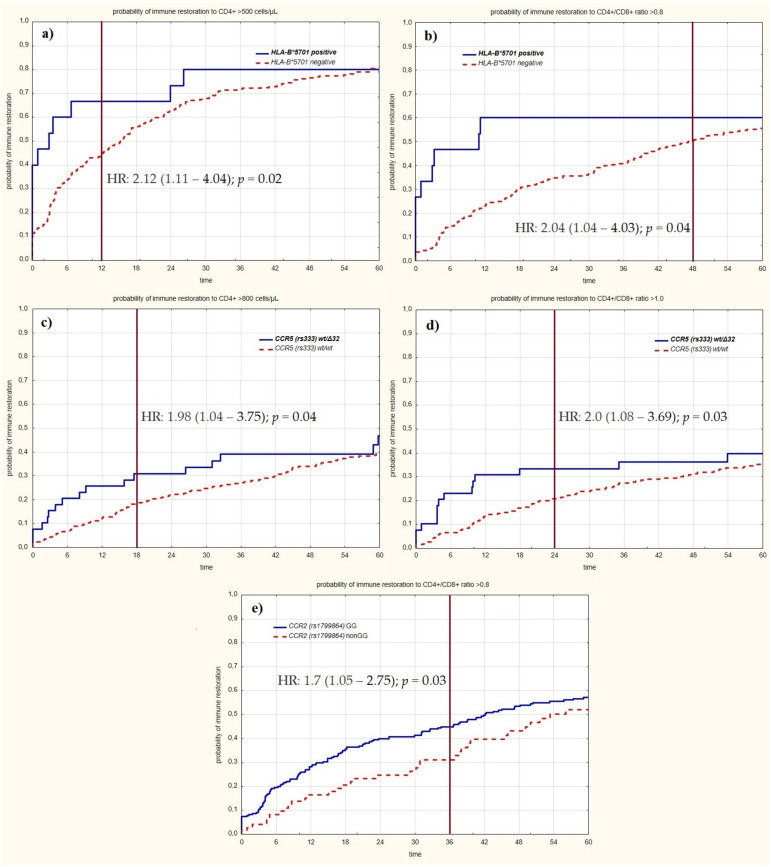
Probability of immune restoration to different points of immune restoration. A vertical line represents the last significant HR. (**a**) of HLA-B*5701 to >500 cells/μL. (**b**) of HLA-B*5701 to CD4+/CD8+ ratio >0.8. (**c**) of CCR5 (rs 333) Δ32/wt to >800 cells/μL. (**d**) of CCR5 (rs 333) Δ32/wt to CD4+/CD8+ ratio >1.0. (**e**) of CCR2 (rs 1799854) GG to CD4+/CD8+ ratio >0.8.

Initially, we analyzed the influence of selected genetic variants at 6 monthly intervals until the 120-month observation timepoint. However, in all cases, significance effects were lost within 48 months (Table 1, Figure 1). For the first 12 months following cART initiation, CCR2 (rs1799864) GG homozygosity was associated with a higher likelihood of immune recovery to a CD4+ cell count >800 cells/μL (hazard ratio (HR): 1.87 (IQR: 1.01–3.45), *p* = 0.05). In addition, the CCR5 wt/Δ32 genotype positively influenced immune recovery to a CD4+ cell count >800 cells/μL (HR: 2.32 (IQR:1.14–4.73), *p* = 0.02) and a CD4+/CD8+ cell ratio >1.0 (HR: 2.62 (IQR: 1.36–5.05), *p* = 0.004). Similarly, the presence of the HLA-B*5701 allele was associated with a higher likelihood of immune restoration to a CD4+ cell count >500 cells/μL, and to a CD4+/CD8+ cell ratio >0.8, with HRs of 2.13 (IQR: 1.1–4.04, *p* = 0.02) and 3.75 (IQR: 1.86–7.54, *p* < 0.001), respectively (Supplementary Table S1).

A notable influence of the HLA-B, CCR2, and CCR5 genetic variants on immunoreactivity was observed for the first year after cART initiation. This effect was lost after 1–4 years. For HLA-B*57, the effect on the CD4/CD8 ratio was lost within 48 months (HR = 2.04, 95% CI: 1.04–4.03), and the effect on the CD4+ cell count was lost within 12 months (HR = 2.12, CI: 1.11–4.04). The effect of CCR2 GG on the CD4+/CD8+ ratio was lost within 36 months (HR = 1.7, CI: 1.05–2.75). For CCR5 wt/Δ32, the effect on the CD4+/CD8+ ratio was lost within 24 months (HR = 2.0, CI: 1.08–3.69), and the effect on CD4+ cell count was lost within 18 months (HR = 1.98, CI: 1.14–4.73) (Table 1).

### 3.3. Clinical Factors Associated with Immune Recovery

**Figure 2:**
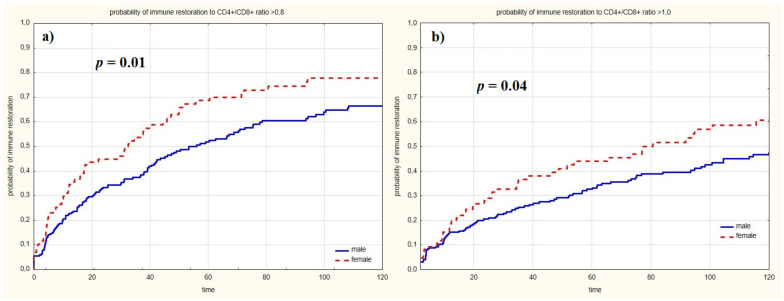
Influence of sex on immune restoration. (**a**) to CD4+/CD8+ ratio >0.8. (**b**) to CD4+/CD8+ ratio >1.0.

As detailed above, the analysis of clinical data related to immune restoration was assessed until the 120-month endpoint (Table 2). Women more commonly showed immunological recovery than men to a CD4+/CD8+ cell ratio >0.8 (72.1% vs. 58.4%, respectively; *p* = 0.01) and >1.0 (54.7% vs. 42%, respectively; *p* = 0.04). The Kaplan–Meier analysis demonstrated that immune recovery at the 120-month timepoint was dependent on sex (Figure 2).

The median age of patients who showed immune recovery was notably lower for all analyzed endpoints, except for the CD4+/CD8+ ratio >1.0. The median age of those who recovered to a CD4+ lymphocyte count >500 cells/μL was 31.5 (IQR: 26–39) years compared with 37 (IQR: 32–44) years for patients without recovery (*p* = 0.001). The median age of those who recovered to a CD4+ lymphocyte count >800 cells/μL was 30 (IQR: 26–39) years compared with 34 (IQR: 27–41) years for those without such recovery (*p* = 0.02), whereas the median age of those who recovered to a CD4+/CD8+ cell ratio >0.8 was 31 (IQR: 26–38) years compared with 34 (IQR: 27–43) years for those who never achieved this threshold (*p* = 0.04).

As expected, the clinical category at presentation significantly affected immunological recovery. Asymptomatic infection at diagnosis was more frequent in patients who reconstructed the CD4+ lymphocyte count to >500 cells/μL (48.2% vs. 20%) and to >800 cells/μL (61.1% vs. 38.9%), and in those who recovered their CD4+/CD8+ cell ratio to >0.8 (74.3% vs. 25.7%) or > 1.0 (62.8% vs. 37.2%; *p* < 0.001).

The HIV subtype notably influenced immunological recovery. Patients infected with non-B variants less frequently achieved CD4+ lymphocyte levels >800 cells/μL (73.6% vs. 26.4%; *p* = 0.003). Additionally, patients who recovered to CD4+ lymphocyte levels >800 cells/μL presented with lower viral loads at baseline compared to patients who did not recover, with median viral loads of 4.78 (4.15–5.32) vs. 5.04 (4.35–5.65) log copies/mL, respectively (*p* = 0.003).

### 3.4. Multivariate Model of Immune Recovery

The final analysis performed in the current study was a multivariate analysis of all immunological endpoints (CD4+ cell count >500 and >800 cells/μL, and CD4+/CD8+ cell ratio >0.8 and >1.0) at the 12-month timepoint using a Cox proportional hazard model. At this timepoint, most of the selected genetic variants were found to have significant effects, as noted above.

**Figure 3:**
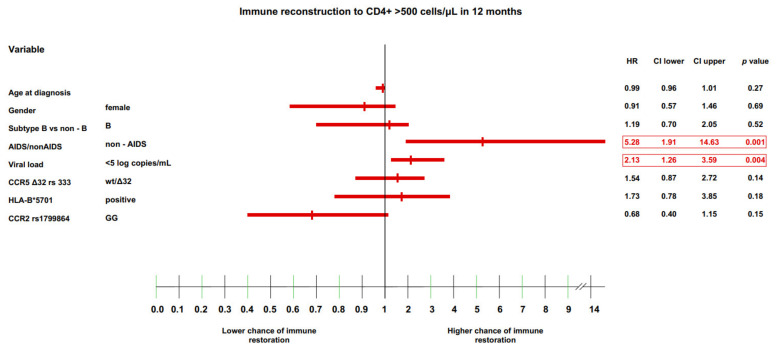
Immune recovery to CD4 >500cells/μL in 12 months.

For the CD4+ lymphocytes >500 cells/μL endpoint, the variables that remained statistically significant throughout the analysis were: (i) no history of AIDS at baseline (HR: 5.28 (IQR: 1.91–14.63), *p* = 0.001) and (ii) a viral load <5 log copies/mL (HR: 2.13 (IQR: 1.26–3.59), *p* = 0.004; (Figure 3).

**Figure 4:**
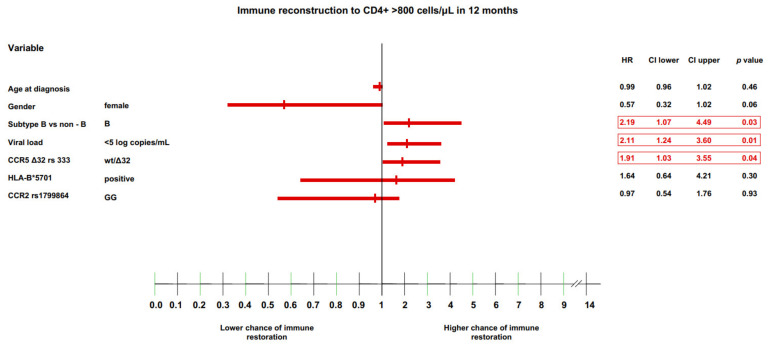
Immune recovery to CD4 >800cells/μL in 12 months. For AIDS/non-AIDS data missing due to insufficient data to calculate HR, the data were omitted.

For the CD4+ lymphocytes >800 cells/μL endpoint, the factors that were significantly associated with a higher likelihood of immune recovery were: (i) HIV subtype B (HR: 2.19 (IQR: 1.07–4.49), *p* = 0.03), (ii) baseline HIV viremia <5 log copies/mL (HR: 2.11 (IQR: 1.24–3.6), *p* = 0.01), and (iii) CCR5 Δ32/wt heterozygosity (HR: 1.91 (IQR: 1.03–3.55), *p* = 0.04; (Figure 4).

**Figure 5:**
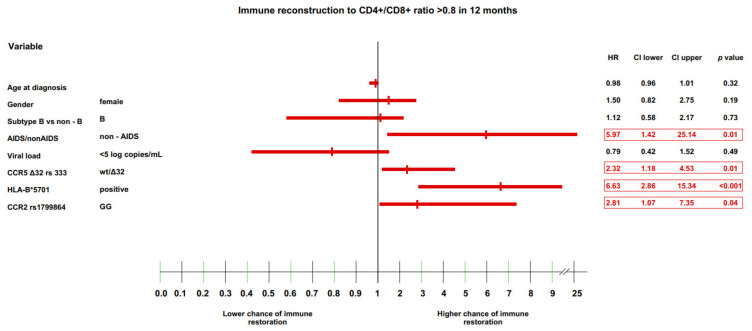
Immune recovery to CD4+/CD8+ ratio >0.8 in 12 months.

The variables that were significantly associated with a CD4+/CD8+ cell ratio >0.8 were: (i) the lack of AIDS at care entry (HR: 5.97 (IQR: 1.42–25.14), *p* = 0.01), (ii) CCR5 Δ32/wt heterozygosity (HR: 2.32 (IQR: 1.18–4.53), *p* = 0.010), (iii) the presence of the HLA-B*5701 allele (HR: 6.63 (IQR: 2.86–15.34), *p* < 0.001), and (iv) the CCR2 GG genotype (HR: 2.81 (IQR: 1.07–7.35), *p* = 0.04); (Figure 5).

**Figure 6:**
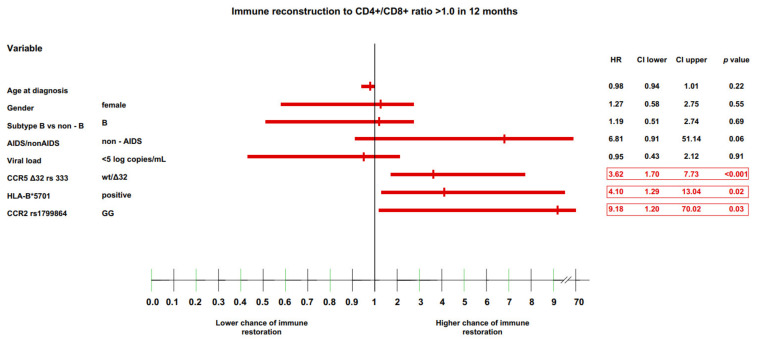
Immune recovery to CD4+/CD8+ ratio >1.0 in 12 months.

Only the genetic factors, CCR5 wt/Δ32 genotype (HR: 3.62 (IQR: 1.70–7.73), *p* < 0.001), the HLA-B*5701 allele (HR: 4.1 (IQR: 1.29–13.04), *p* = 0.02), and the CCR2 GG genotype (HR: 9.18 (IQR: 1.20–70.02), *p* = 0.03) were significantly associated with a CD4+/CD8+ cell ratio >1.0 (Figure 6).

## 4. Discussion

Factors that increase the likelihood of immune recovery may be divided into clinical factors, such as sex and clinical category at the time of diagnosis; virological factors, such as the HIV subtype or viremia at diagnosis; and genetic factors, including HLA-B and chemokine gene variants. The novel data presented here show that genetic variants significantly affect the immune recovery of HIV-infected patients. We also found that these variants had the greatest impact at the beginning of the observation period which is also the time point of treatment initiation.

A CD4+ lymphocyte count of 500 cells/μL has traditionally been treated as a target point for immune restoration. This target is a direct result of the division into historical immunological categories, as suggested by the CDC as early as 1993 [30]. However, several guidelines use the CD4+ lymphocyte immune recovery point of >800 cells/μL as reflective of a more complete immune recovery [26]. This revised target is also associated with a lower risk of cardiovascular-disease-related death, and it was assessed in other studies of immune restoration [31,32]. A CD4+/CD8+ cell ratio >1.0 was used as it was observed in uninfected individuals and is considered to represent a complete immune response. A CD4+/CD8+ cell ratio >0.8 is an intermediate recovery value. In the Aquitaine cohort, this ratio was found to be associated with a lower risk of bacterial infections in HIV-infected patients [33].

In the current study, the factors that had a positive impact on immunological restoration were a lower age at diagnosis, which may be associated with the longer follow-up time and greater regenerative capacity in young patients. Similar findings were observed in many other studies, where a lower age at diagnosis was shown to influence immune recovery positively [4,34,35,36,37].

The clinical category at the time of entry into care was also expected to be associated with a higher probability of immune restoration. This is related to the higher baseline CD4+ cell counts in asymptomatic patients. These patients were most likely infected for a relatively short time, and therefore, they are unlikely to have significant disease progression and, consequently, a decrease in the baseline CD4+ lymphocyte count [1]. The assessment of immune restoration in HIV-infected individuals shows that higher CD4+ cell counts at the time of the initiation of ART result in a greater chance of rebuilding this lymphocyte population to normal levels [27,38].

HIV subtype B was found to be associated with more frequent immune restoration of CD4 lymphocyte counts to >800 cells/µL compared to non-B variants. This was not confirmed in previous international studies [39,40,41]. This finding may be related to the dominant HIV-1 subtype B route of transmission, which is men having sex with men [42]. This route of transmission has been widely examined, and this population is often tested for HIV; therefore, they enter care at an early stage of infection, and they also have higher baseline CD4+ cell counts. It should also be noted that in previous studies in our center, subtype D was found to be associated with more severe immune destruction, later diagnosis, and higher viral load upon entry into care, which is consistent with the analysis presented herein [43,44]. However, the influence of HIV subtype on immune restoration was not confirmed in the multivariate analyses in this study.

We also found that people with HIV-1 viremia <5 log copies/mL at the time of diagnosis more often achieved immune restoration to >800 cells/µL. This is consistent with the observations from the Swiss cohort and the Asia Pacific HIV Observational Database cohorts, in which patients with higher baseline HIV viral load reconstructed immunologically to lower CD4+ cell counts [36,45].

In the context of genetic factors, the HLA-B57*01 variant was associated with a significantly shorter time required for immune recovery. This increases the chances of recovery to both the number of CD4+ lymphocytes >500 cells/µL and to a CD4+/CD8+ cell ratio >0.8. These effects of the HLA-B57*01 variant on the baseline number of CD4+ lymphocytes and the CD4+ lymphocyte nadir were previously demonstrated [24]. Other studies also showed a positive effect of the HLA-B57*01 variant on the delay of disease progression, virological control, and the response to antiretroviral therapy [17,46,47,48,49].

The positive effect of the GG genotype of the CCR2 gene on immune restoration to a CD4+/CD8+ cell ratio >0.8 is reported infrequently, despite numerous previous clinical studies. Restrepo et al. showed a negative effect of the CCR2 gene AG genotype on immune restoration to >200 CD4+ cells/µL after 2 years on cART and suppressed viral replication, especially in combination with the TT genotype of rs1801157 in the CXCL12 gene [50]. Similarly, in a Chinese cohort, the CCR2 A allele was not protective against the acquisition of HIV-1 infection, but this variant was shown to influence the clinical category, with a predominance of symptomatic infections [51]. On the contrary, Passam et al. showed a positive effect of the CCR2 A allele on the time taken to obtain undetectable viremia in patients undergoing cART [52]. Patients with the A allele obtained undetectable viremia in 3.5 ± 0.48 months compared to 10.26 ± 1.42 months for those with the G allele.

Another variant that was shown to have a positive effect on immune restoration was the heterozygous 32 bp deletion in the CCR5 gene. This effect was observed in relation to immune restoration to both CD4+ lymphocytes >800 cells/µL and the CD4+/CD8+ cell ratio >1.0. Many previous studies show a beneficial effect of this variant on the number of CD4+ lymphocytes, the rate of immune recovery, and protection against AIDS [53,54,55]. It was also suggested that this variant has a beneficial effect throughout the entire infection period [8], which was not demonstrated in the present study. This may be related to the rapid implementation of treatment in the studied cohort and the dominant beneficial effect of cART treatment on patient survival.

In the current study, the influence of genetic variants on immune restoration was found to be strongest at the beginning of the observation period (in the first year after diagnosis) and then decreased with time, losing statistical significance at the fifth year of observation. A similar phenomenon was observed in other studies analyzing the influence of genetic variants on HIV progression, in which the period of observation rarely extended beyond 2 years [50,54].

## 5. Conclusions

Immune recovery in HIV-infected patients allows for a lower risk of exposure to opportunistic diseases, comorbidities, and death. In addition to typical infectious agents, a normalized immunological state may have an impact on the development of cardiovascular disease or neoplasms unrelated to AIDS. Analyses of the influence of clinical and genetic factors on immunological recovery provide a greater understanding of the mechanisms involved in this process. They provide information to help assess groups of patients who are at risk of incomplete immune recovery in the future.

Clinical parameters, such as a younger age, asymptomatic infection, or lack of AIDS (according to the CDC 1993 case definition); female sex; HIV subtype B; viremia lower than 5 log copies/mL; and genetic parameters, such as the presence of the HLA-B*5701 allele, heterozygosity at rs333 in CCR5, and GG homozygosity at rs1799864 in CCR2 increased the likelihood of immune restoration. Genetic factors had a greater influence at the beginning of the observation period in HIV-infected patients, losing their clinical significance over time.

### Study Limitations

Among the analyzed data, there were none relating to comorbidities. Moreover, the time to diagnosis of HIV was not assessed—no data were collected that could be used to calculate the time to diagnosis of HIV.

## Figures and Tables

**Table 1:** Expiry time of statistical significance of selected genetic variants for immune recovery.

	HR (95% CI) (12 Months)	*p* (Cox Regression)	Number (%) of Patients Who Reconstructed (Kaplan–Meier Estimator)		HR (95% CI) (18 Months)	*p* (Cox Regression)	Number (%) of Patients Who Reconstructed (Kaplan–Meier Estimator)		HR (95% CI) (24 Months)	*p* (Cox Regression)	Number (%) of Patients Who Reconstructed (Kaplan–Meier Estimator)		HR (95% CI) (30 Months)	*p* (Cox Regression)	Number (%) of Patients Who Reconstructed (Kaplan–Meier Estimator)		HR (95% CI) (36 Months)	*p* (Cox Regression)	Number (%) of Patients Who Reconstructed (Kaplan–Meier Estimator)		HR (95% CI) (42 Months)	*p* (Cox Regression)	Number (%) of Patients Who Reconstructed (Kaplan–Meier Estimator)		HR (95% CI) (48 Months)	*p* (Cox Regression)	Number (%) of Patients Who Reconstructed (Kaplan–Meier Estimator)	
HLA-B*5701 immune recovery to CD4/CD8 ratio >0.8 (number of patients 265)																												
positive	3.74 (1.84–7.54)	*p* < 0.001	6 (40.00%)	*p* < 0.001	3.47 (1.73–6.95)	*p* < 0.001	9 (64.29%)	*p* = 0.002	3.02 (1.52–6.0)	*p* = 0.001	9 (64.29%)	*p* = 0.008	2.89 (1.46–5.75)	*p* = 0.002	9 (64.29%)	*p* = 0.01	2.59 (1.31–5.12)	*p* = 0.006	9 (64.29%)	*p* = 0.02	2.24 (1.13–4.41)	*p* = 0.02	9 (64.29%)	*p* = 0.06	2.04 (1.04–4.03)	*p* = 0.04	9 (64.29%)	*p* = 009
negative	ref.		63 (23.08%)		ref.		75 (29.88%)		ref.		88 (35.06%)		ref.		92 (36.65%)		ref.		103 (41.04%)		ref.		118 (47.01%)		ref.		127 (50.60%)	
CCR2 (rs1799864) immune recovery to CD4/CD8 ratio >0.8 (number of patients 277)																												
GG	1.87 (1.01–3.45)	*p* = 0.05	65 (28.14%)	*p* = 0.04	2.0 (1.11–3.61)	*p* = 0.02	76 (35.85%)	*p* = 0.01	1.87 (1.1–3.19)	*p* = 0.02	86 (40.57%)	*p* = 0.02	1.83 (1.09–3.07)	*p* = 0.02	89 (41.98%)	*p* = 0.02	1.7 (1.05–2.75)	*p* = 0.03	97 (45.75%)	*p* = 0.02	in this time interval, the data were not statistically significant							
nonGG	ref.		12 (16.44%)		ref.		13 (20.00%)		ref.		16 (24.62%)		ref.		17 (26.15%)		ref.		20 (30.77%)									
CCR5 Δ32 (rs333) immune recovery to CD4/CD8 ratio >1.0 (number of patients 276)																												
wt/Δ32	2.62 (1.36–5.05)	*p* = 0.003	12 (30.77%)	*p* = 0.004	2.44 (1.3–4.56)	*p* = 0.005	13 (34.21%)	*p* = 0.007	2.0 (1.08–3.69)	*p* = 0.03	13 (34.21%)	*p* = 0.03	in this time interval, the data were not statistically significant															
wt/wt	ref.		35 (13.41%)		ref.		39 (16.39%)		ref.		48 (20.17%)																	
CCR5 Δ32 (rs333) immune recovery to CD4+ >800 cells/µL (number of patients 276)																												
wt/Δ32	2.32 (1.14–4.73)	*p* = 0.02	10 (25.64%)	*p* = 0.02	1.98 (1.04–3.75)	*p* = 0.04	12 (31.58%)	*p* = 0.04	in this time interval, the data were not statistically significant																			
wt/wt	ref.		32 (12.26%)		ref.		43 (18.07%)																					
HLA-B*5701 immune recovery to CD4+ >500 cells/µL (number of patients 287)																												
positive	2.12 (1.11–4.04)	*p* = 0.02	10 (66.67%)	*p* = 0.04	in this time interval, the data were not statistically significant																							
negative	ref.		122 (44.85%)																									

**Table 2:** Immunological recovery in patients treated with cART in 120 months.

	Immunological Recovery >500 Cells/µL			Immunological Recovery >800 Cells/µL			Immunological Recovery of CD4+/CD8+ Ratio >0.8			Immunological Recovery of CD4+/CD8+ Ratio >1.0		
	No	Yes		No	Yes		No	Yes		No	Yes	
Sex												
female, *n* = 86 (%)	10 (11.6%)	76 (88.4%)	*p* = 0.22	48 (55.8%)	38 (44.2%)	*p* = 0.57	24 (27.9%)	62 (72.1%)	*p* = 0.02	39 (45.3%)	47 (54.7%)	*p* = 0.05
male, *n* = 226 (%)	39 (17.3%)	187 (82.7%)		118 (52.2%)	108 (47.8%)		94 (41.6%)	132 (58.4%)		131 (58%)	95 (42%)	
median age at diagnosis (IQR)	37 (32–44)	31.5 (26–39)	*p* = 0.001	34 (27–41)	30 (26–39)	*p* = 0.02	34 (27–43)	31 (26–38)	*p* = 0.04	34 (27–42)	31 (27–38)	*p* = 0.08
Likely mode of transmission												
MSM, *n* = 114 (%)	13 (11.4%)	101 (88.6%)	*p* = 0.72	51 (44.7%)	63 (55.3%)	*p* = 0.46	41 (36%)	73 (64%)	*p* = 0.93	64 (56.1%)	50 (43.9%)	*p* = 0.91
HSX, *n* = 98 (%)	14 (14.3%)	84 (85.7%)		55 (56.1%)	43 (43.9%)		37 (37.8%)	61 (62.2%)		49 (50%)	49 (50%)	
IDU, *n* = 56 (%)	11 (19.6%)	45 (80.4%)		32 (57.1%)	24 (42.9%)		21 (37.5%)	35 (62.5%)		28 (50%)	28 (50%)	
Clinical category at diagnosis (according to CDC from 1993 y.)												
Asymptomatic, *n* = 112 (%)	8 (20%)	104 (47.9%)	*p* < 0.001	44 (33.1%)	68 (54.8%)	*p* < 0.001	28 (30.1%)	84 (51.2%)	*p* < 0.001	41 (30.6%)	71 (57.7%)	*p* < 0.001
Symptomatic-non-AIDS, *n* = 95 (%)	15 (37.5%)	80 (36.9%)		53 (39.8%)	42 (33.9%)		34 (36.6%)	61 (37.2%)		54 (40.3%)	41 (33.3%)	
AIDS defining illness, *n* = 50 (%)	17 (42.5%)	33 (15.2%)		36 (27.1%)	14 (11.3%)		31 (33.3%)	19 (11.6%)		39 (29.1%)	11 (8.9%)	
HIV subtype												
B, *n* = 154 (%)	23 (14.9%)	131 (85.1%)	*p* = 0.06	77 (50%)	77 (50%)	*p* = 0.003	56 (36.4%)	98 (63.6%)	*p* = 0.65	83 (53.9%)	71 (46.1%)	*p* = 0.41
Non-B, *n* = 53 (%)	14 (26.4%)	39 (73.6%)		39 (73.6%)	14 (26.4%)		21 (39.6%)	32 (60.4%)		32 (60.4%)	21 (39.6%)	
Virological characteristics												
Median viral load at diagnosis (IQR) (log copies/mL)	5.07 (4.81–5.54)	4.9 (5.52–6.31)	*p* = 0.13	5.04 (4.35–5.65)	4.78 (4.15–5.32)	*p* = 0.003	5.0 (4.11–5.42)	4.81 (4.27–5.42)	*p* = 0.06	4.99 (4.2–5.59)	4.79 (4.18–5.18)	*p* = 0.07
Viral load <5log copies/mL, *n* = 154 (%)	17 (11%)	137 (89%)	*p* = 0.06	52 (38%)	85 (62%)	*p* = 0.03	35 (25.5%)	102 (74.5%)	*p* = 0.22	60 (43.8%)	77 (56.2%)	*p* = 0.36
Viral load >5 log copies/mL, *n* = 144 (%)	27 (18.8%)	117 (81.3%)		60 (51.3%)	57 (48.7%)		38 (32.5%)	79 (67.5%)		58 (49.6%)	59 (50.4%)	

## Data Availability

The data presented in this study are available on request from the corresponding author. The data are not publicly available due to privacy restrictions.

## Supplementary Materials

The following supporting information can be downloaded at: https://www.mdpi.com/article/10.3390/jcm11071887/s1, Table S1: Immune recovery in patients treated with cART within 12 months.
