# Supplementary material for: Factors Influencing Immune Restoration in People Living with HIV/AIDS
Source: J Clin Med. 2022 Mar 28;11(7):1887. doi: 10.3390/jcm11071887 (PMC9000185; doi:10.3390/jcm11071887)
Supplement: Supplementary file 1 [file jcm-11-01887-s001.zip › jcm-1615867-supplementary.pdf]

Journal of
Clinical Medicine

MDPI

Supplementary Material: Factors Influencing Immune Restoration in People Living with HIV/AIDS

Bogusz Jan Aksak-Wąs, Anna Urbańska, Kaja Scheibe, Karol Serwin, Magdalena Leszczyszyn-Pynka, Milena Rafalska-Kosior, Joanna Gołąb, Daniel Chober and Miłosz Parczewski

Table S1. Immune recovery in patients treated with cART within 12 months.

	HR (95% CI) (CD4+ >500 cells/µL)	*p* (Cox regression)	lower 95% confidence interval	upper 95% confidence interval	number (%) of patients who reconstructed to CD4 +> 500 cells/µL (Kaplan–Meier estimator)		HR (95% CI) (CD4+ >800 cells/µL)	p (Cox regression)	lower 95% confidence interval	upper 95% confidence interval	number (%) of patients who reconstructed to CD4 +> 800 cells/µL (Kaplan–Meier estimator)log-rank		HR (95% CI) (CD4+/CD8+ ratio >0.8)	p (Cox regression)	lower 95% confidence interval	upper 95% confidence interval	number (%) of patients who reconstructed to CD4+/CD8 ratio >0.8 (Kaplan–Meier estimator)log-rank		HR (95% CI) (CD4+/CD8+ ratio >1.0)	p (Cox regression)	lower 95% confidence interval	upper 95% confidence interval	number (%) of patients who reconstructed to CD4+/CD8 ratio >1.0 (Kaplan–Meier estimator)log-rank	
CCR2 rs1799864 (number of patients 303)																								
AA	1.43	*p* = 0.54	0.45	4.48	3 (60.00 %)	*p* = 0.55	no possibility to define HR				0 (0.00%)	*p* = 0.36	no possibility to define HR				0 (0.00%)	*p* = 0.19	no possibility to define HR				0 (0.00%)	*p* = 0.33
non-AA	ref.				135 (45.30 %)						43 (14.43 %)						77 (25.75 %)						48 (16.11 %)	
AG	ref.	*p* = 0.99	0.67	1.5	31 (45.59 %)	*p* = 0.99	1.18	*p* = 0.63	0.6	2.35	11 (16.18 %)	*p* = 0.63	ref.	*p* = 0.1	0.91	3.12	12 (17.65 %)	*p* = 0.08	ref.	*p* = 0.16	0.8	3.96	7 (10.29 %)	*p* = 0.15
non-AG	1.0				107 (45.53 %)		ref.				32 (13.62 %)		1.69				65 (27.54 %)		1.78				41 (17.45 %)	
GG	ref.	*p* = 0.87	0.7	1.52	104 (45.22 %)	*p* = 0.88	ref.	*p* = 0.84	0.54	2.13	32 (13.91 %)	*p* = 0.84	1.87	*p* = 0.05	1.01	3.45	65 (28.14 %)	*p* = 0.04	1.96	*p* = 0.1	0.88	4.36	41 (17.83 %)	*p* = 0.09
non-GG	1.03				34 (46.58 %)		1.07				11 (15.07 %)		ref.				12 (16.44 %)		ref.				7 (9.59%)	
CCR5 promoter, rs1799988 (number of patients 302)																								
CC	1.03	*p* = 0.85	0.73	1.46	49 (46.23 %)	*p* = 0.85	1.1	*p* = 0.76	0.59	2.05	16 (15.09 %)	*p* = 0.76	ref.	*p* = 0.36	0.77	2.03	24 (22.43 %)	*p* = 0.36	1.11	*p* = 0.74	0.62	1.99	18 (16.98 %)	*p* = 0.72
non-CC	ref.				89 (45.41 %)		ref.				27 (13.78 %)		1.25				53 (27.04 %)		ref.				30 (15.31 %)	
CT	1.06	*p* = 0.75	0.76	1.47	67 (46.85 %)	*p* = 0.74	1.3	*p* = 0.39	0.72	2.37	23 (16.08 %)	*p* = 0.38	ref.	*p* = 0.96	0.65	1.58	36 (25.17 %)	*p* = 0.95	ref.	*p* = 0.84	0.6	1.87	22 (15.38 %)	*p* = 0.84
non-CT	ref.				71 (44.65 %)		ref.				20 (12.58 %)		1.01				41 (25.63 %)		1.06				26 (16.35 %)	
TT	ref.	*p* = 0.52	0.74	1.83	22 (41.51 %)	*p* = 0.51	ref.	*p* = 0.14	0.77	6.06	4 (7.55%)	*p* = 0.12	1.41	*p* = 0.21	0.82	2.41	17 (32.08 %)	*p* = 0.22	ref.	*p* = 0.86	0.5	2.29	8 (15.09 %)	*p* = 0.86
non-TT	1.16				116 (46.59 %)		2.17				39 (15.66 %)		ref.				60 (24.00 %)		1.07				40 (16.06 %)	
CX3CR1 rs3732378 (number of patients 303)																								

AA	ref.	$p = 0.77$	0.52	2.39	7 (43.75%)	$p = 0.76$	ref.	$p = 0.86$	0.28	4.72	2 (12.50%)	$p = 0.85$	ref.	$p = 0.59$	0.43	4.36	3 (18.75%)	$p = 0.58$	ref.	$p = 0.76$	0.3	5.16	2 (12.50%)	$p = 0.76$
non-AA	1.12				131 (45.64%)		1.14				41 (14.29%)		1.38				74 (25.69%)		1.25				46 (16.03%)	
AG	ref.	$p = 0.25$	0.86	1.78	42 (41.58%)	$p = 0.23$	ref.	$p = 0.14$	0.85	3.48	10 (9.90%)	$p = 0.13$	ref.	$p = 0.9$	0.64	1.66	25 (24.75%)	$p = 0.9$	1.34	$p = 0.33$	0.75	2.38	19 (18.81%)	$p = 0.33$
non-AG	1.24				96 (47.52%)		1.72				33 (16.34%)		1.03				52 (25.62%)		ref.				29 (14.36%)	
GG	1.25	$p = 0.21$	0.88	1.77	89 (47.85%)	$p = 0.19$	1.68	$p = 0.13$	0.86	3.28	31 (16.67%)	$p = 0.12$	1.09	$p = 0.71$	0.69	1.74	49 (26.20%)	$p = 0.71$	ref.	$p = 0.42$	0.72	2.24	27 (14.52%)	$p = 0.42$
non-GG	ref.				49 (41.88%)		ref.				12 (10.26%)		ref.				28 (23.93%)		1.27				21 (17.95%)	
HLA-C -35 rs9264942 (number of patients 293)																								
CC	ref.	$p = 0.59$	0.71	1.81	21 (42.00%)	$p = 0.58$	ref.	$p = 0.56$	0.55	3.07	6 (12.00%)	$p = 0.55$	ref.	$p = 0.63$	0.62	2.22	11 (22.00%)	$p = 0.63$	ref.	$p = 0.24$	0.69	4.43	5 (10.00%)	$p = 0.22$
non-CC	1.14				113 (46.50%)		1.3				36 (14.81%)		1.17				63 (25.82%)		1.75				41 (16.87%)	
TT	1.05	$p = 0.8$	0.74	1.48	52 (46.43%)	$p = 0.79$	1.68	$p = 0.09$	0.92	3.08	21 (18.75%)	$p = 0.09$	ref.	$p = 0.55$	0.72	1.86	26 (23.21%)	$p = 0.55$	1.04	$p = 0.91$	0.57	1.87	18 (16.07%)	$p = 0.91$
non-TT	ref.				82 (45.30%)		ref.				21 (11.60%)		1.16				48 (26.37%)		ref.				28 (15.47%)	
CCR5 Δ32 rs 333 (number of patients 300)																								
wt/wt	ref.	$p = 0.26$	0.82	2.11	116 (44.44%)	$p = 0.27$	ref.	$p = 0.02$	1.14	4.73	32 (12.26%)	$p = 0.02$	ref.	$p = 0.09$	0.93	2.96	62 (23.66%)	$p = 0.09$	ref.	$p = 0.004$	1.36	5.05	35 (13.41%)	$p = 0.004$
wt/Δ32	1.31				20 (51.28%)		2.32				10 (25.64%)		1.66				14 (35.90%)		2.62				12 (30.77%)	
HLA-B*5701 (number of patients 287)																								
positive	2.13	$p = 0.02$	1.1	4.04	10 (66.67%)	$p = 0.04$	ref	$p = 1.0$	0.24	4.15	2 (13.33%)	$p = 1.0$	3.75	$p < 0.001$	1.86	7.54	9 (60.00%)	$p < 0.001$	1.86	$p = 0.24$	0.67	5.19	4 (26.67%)	$p = 0.24$
negative	ref.				122 (44.85%)		1.0				38 (13.97%)		ref.				63 (23.08%)		ref.				42 (15.44%)	
